# Sex-Differences in Pain and Opioid Use Disorder Management: A Cross-Sectional Real-World Study

**DOI:** 10.3390/biomedicines10092302

**Published:** 2022-09-16

**Authors:** Mónica Escorial, Javier Muriel, César Margarit, Laura Agulló, Domingo Morales, Ana M. Peiró Peiró

**Affiliations:** 1 Neuropharmacology Applied to Pain (NED), Alicante Institute for Health and Biomedical Research (ISABIAL), c/Pintor Baeza, 12, 03010 Alicante, Spain; 2 Institute of Bioengineering, Miguel Hernández University, Avda. de la Universidad s/n, 03202 Elche, Spain; 3 Pain Unit, Dr. Balmis General University Hospital, ISABIAL, c/Pintor Baeza, 12, 03010 Alicante, Spain; 4 Operations Research Centre, Miguel Hernández University, Avda. de la Universidad s/n, 03202 Elche, Spain; 5 Clinical Pharmacology Department, Dr. Balmis General University Hospital, ISABIAL, c/Pintor Baeza, 12, 03010 Alicante, Spain

**Keywords:** opioid use disorder, sex-differences, chronic non-cancer pain, gender disparities, pain management, prevention programs

## Abstract

(1) Background: It is essential to focus attention on sex-specific factors which are clinically relevant in pain management, especially with regards to opioid use disorder (OUD) risk. The aim of this study was to explore potential sex-differences in chronic non-cancer pain (CNCP) outpatients. (2) Methods: An observational cross-sectional study was conducted under CNCP outpatients with long-term prescribed opioids (*n* = 806), wherein 137 patients had an OUD diagnosis (cases, 64% females) and 669 did not (controls, 66% females). Socio-demographic, clinical, and pharmacological outcomes were analyzed. (3) Results: Female controls presented an older age and less intensive pain therapy but higher psychotropic prescriptions and emergency department visits compared to male controls. Meanwhile, cases demonstrated a younger age, higher work disability, double morphine equivalent daily dose, and benzodiazepine use compared with controls. Here, female cases showed an 8% greater substance use disorder (OR 2.04 [1.11–3.76]) and 24% lower tramadol use, while male cases presented a 22% higher fentanyl use (OR 2.97 [1.52–5.81]) and reported the highest number of adverse drug reactions (24%, OR 2.40 [1.12–5.16]) compared with controls. (4) Conclusions: An OUD individual risk profile was evidenced with sex-differences to take into consideration to design equal prevention programs.

## 1. Introduction

Chronic pain is one of the leading causes of medical consultation among adults and the main cause of abandonment of their daily activity [1]. It is estimated that 19% of Europeans suffer from chronic pain [2], resulting in important emotional, social, and economic consequences for the patient and his/her environment. Likewise, it is a major health problem associated with great costs and consumption of health resources (around 2.5% of the Spanish GDP) [3]. Normally, it is accompanied by a wide range of comorbidities and risk factors for other adverse health outcomes [4]. It has been demonstrated that women are more vulnerable for developing and maintaining musculoskeletal pain than men [5,6]. Findings from the literature suggest that women are more likely to be prescribed opioids for non-medical use [7], often with higher emotional and affective distress [8] compared with men. As opioid prescription is the usual therapy for CNCP, the question can be raised: Are females at a different risk for developing an opioid use disorder (OUD) than men?

Whilst it is important to clearly distinguish between sex and gender, we also need to understand the mechanisms and pathways underlying the trends we observe, as well as how sex and gender intersect with other factors such as age, income, social status, education, employment, genetics, or personal health practice, and contribute to our health and overall health outcome [9]. There is limited information on sex-differences in OUD risk factors. In general, a young age, past or current substance use, untreated psychiatric disorders, preadolescent sexual abuse, and social or family environments that encourage misuse constitute some of the OUD risk factors previously described [10,11,12]. Nevertheless, the limited presence of women in clinical trials and the lack of stratification by sex -mostly restricted to binary comparisons lacking data on gender dynamics raises questions related to sex-differences [13,14]. In this regard, our aim was to identify potential sex-specific risks and needs in CNCP patients using long-term prescribed opioids. The exploratory nature of this study would help to understand sex-differences in OUD risk for a future gender perspective analysis and allow for more equal clinical assessment and treatments.

## 2. Materials and Methods

### 2.1. Study Design

A cross-sectional study was conducted under CNCP outpatients with long-term prescribed opioids (≥6 months) from September 2020 to September 2021 at the Pain Unit (PU) of the Alicante General Hospital. The study is under the umbrella of a master protocol approved by the Ethics Committee of Alicante General Hospital (PI2020-047).

### 2.2. Participants

A total of 137 patients with OUD (cases) were included from an opioid tapering procedure routinely developed at PU [15] under the following inclusion criteria: adults (>18 years old) with CNCP under long-term prescribed opioids (≥6 months) and a clinical diagnosis of OUD Controls data (*n* = 669) were obtained from two concomitant observational studies [16,17] with same inclusion criteria except OUD diagnosis. All variables were collected from their original database and, if needed, they were completed using Electronic Health Records (EHRs), which allows for reviewing medical diagnoses, outcomes, and medication use.

### 2.3. Measures

OUD was diagnosed by a psychiatric expert in pain according to DSM-5 [18] as part of an established opioid tapering procedure [15]. The patient had to meet at least two of the criteria specified in the manual to consider he/she had an OUD.

The independent variable for all of the analysis was the sex of the patient (female/male).

Other socio-demographic characteristics such as age, employment status (active, retired, work disability, unemployed or homemaker) and income (low income as less than €500, middle income as between €500 and 1000, and upper income as more than €1000) were also registered.

A Global Pain State questionnaire [18] measuring, qualitatively, pain, relief, and quality of life was collected at the time of the original interview. Pain intensity and relief were measured using the Visual Analogue Scale (VAS) [19]. Both consist of a horizontal line ranging from 0 (lowest) to 100 mm (highest), where the patient points on the line to the intensity of pain or relief that he/she feels, respectively. Quality of life was evaluated through the EuroQol-5D scale that consists of a VAS (vertical line from 0 (the worst imaginable health status) to 100 mm (the best imaginable) where the patient indicates his/her actual health status. To collect patients’ reports of adverse events (AEs), the most frequent adverse drug reactions (ADRs, selected according to opioids Summary of Product Characteristics frequency as “very common” and “common”) [20], and any other AEs presented, were collected as present/absent. They consisted of the following: sleepiness, dizziness, nausea, vomiting, constipation, itchiness, sexual dysfunction, loss of libido, weight change, headache, skin redness, dry skin, dry mouth, edema, depression, sleep disturbance, nervousness and loss of appetite. In addition, patients were asked about any depression or anxiety symptoms they had. Likewise, all ADRs related to the pain treatment were registered [21]. The presence of history of prior substance use disorder (including tobacco, alcohol and illicit drugs) were registered through the review of medical diagnoses, narratives or any visit to the Addictive Behaviour Unit.

The use (yes/no) of simple analgesics (i.e., paracetamol and metamizole), non-steroidal anti-inflammatory drugs (NSAIDs), and opioids use (i.e., tramadol, codeine, fentanyl, oxycodone, tapentadol, buprenorphine, morphine, hydromorphone and methadone), along with immediate release opioids prescription were registered. In different combinations of opioids, oral morphine equivalent daily dose (MEDD) was estimated using available references [22]. The prescription of antidepressants (i.e., amitriptyline, fluoxetine, escitalopram, and duloxetine), benzodiazepines, and neuromodulators (pregabalin and gabapentin) was also collected.

### 2.4. Statistical Analysis

Convenience sampling was considered based on the prevalence of OUD diagnosis in our regular clinical routine at PU. Data distribution was analysed with the Kolmogorov-Smirnov test using the Lilliefors correction method. Quantitative parametric data are presented as mean (standard deviation (SD)) whilst the median (interquartile range (IQR)) was used for non-parametric data. Categorical data are expressed as percentages (%). Comparisons of socio-demographic, clinical, pharmacological and safety data were evaluated depending upon their distribution. Bivariate odds ratio (OR) and 95% confidence intervals (CIs) were also calculated. Collinearity between categorical variables was tested depending upon their distribution. Here, results were analyzed by groups (men or women, cases vs. controls) or by sex (i.e., control or cases, men vs. women). A *p*-value < 0.05 was considered statistically significant. Analyses were carried out using R (Version 3.2.0; the GNU project, Cambridge, MA, USA) and GraphPad Prism (Version 5.0, Dotmatics, Boston, MA, USA).

## 3. Results

**Figure 1:**
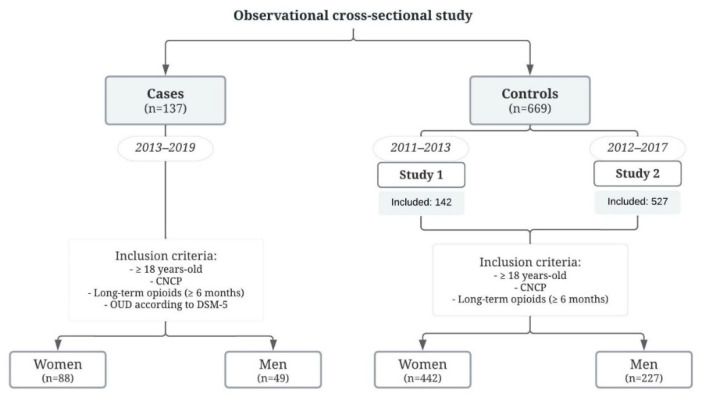
Flow chart of the patient selection for the study. CNCP, chronic non-cancer pain; OUD, opioid use disorder.

A total of 1452 potential control candidates were explored, whereof 783 were excluded due to patients being duplicated between the studies or not meeting the inclusion criteria. All participants included (Figure 1) were referred to our PU for regular pain management mostly due to somatic pain (85%). Non-specific low back pain was the most common type (associated with radiculopathy, spinal stenosis, or another specific spinal cause), followed by knee pain and other musculoskeletal pain (hip pain or due to other cervical joint dysfunctions).

### 3.1. Socio-Demographic and Clinical Outcomes

**Figure 2:**
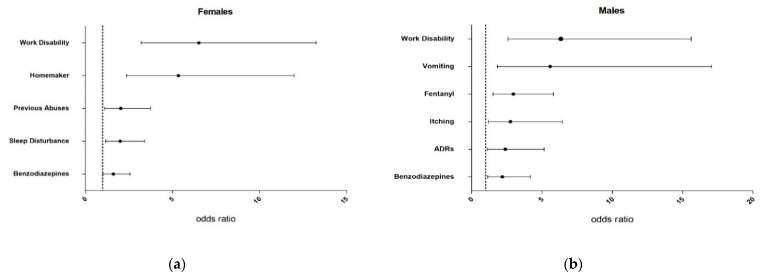
Odds ratio (OR) with 95% confidence intervals (95% CI) of risk factors for females (**a**) and males (**b**). ADRs, adverse drug reactions.

A summary of the characteristics of the participants and clinical variables are shown in Table 1 and Table 2. Meanwhile, Figure 2 shows the odds ratios and 95% confidence intervals for risk factors in females (Figure 2a) and males (Figure 2b).

Controls were older than cases, even more in females (66 (56–75) years old) who were the oldest group (vs. female cases: 53 (45–65) years old, *p* < 0.001; vs. male controls: 53 (45–65), *p* = 0.001). Thus, controls were more retired than cases (females: 56% vs. 22%, *p* < 0.001; males: 53% vs. 28%, *p* = 0.032), whilst the latter presented higher prevalence of work disability (females: 9% vs. 41%, *p* < 0.001, OR 6.52 [3.21–13.27] and males: 22% vs. 64%, *p* = 0.009, OR 6.34 [2.57–15.61]) (Figure 2a,b). Females presented the highest household tasks dedication, even more in cases (27% vs. 7% in controls, *p* < 0.001; OR 5.35 [2.38–12.00]), being 7-times higher than men in both groups. What’s more, a significant 8% greater of SUD was found in female cases relative to controls (19% vs. 11%, *p* = 0.029; OR 2.04 [1.11–3.76]).

### 3.2. Pharmacological Outcomes

Pharmacological outcomes are shown in Table 3 and Table 4.

In control group, sex-differences were observed as females had a greater 8% use of simple analgesics (45% vs. 37% in males, *p* = 0.039), 12% of tramadol (37% vs. 25%, *p* = 0.001), along with greater psychotropic drugs use (11%-antidepressants (42% vs. 31%, *p* = 0.006), 14%-benzodiazepines (41% vs. 27%, *p* < 0.001)) and emergency room visits (32% vs. 22%, *p* = 0.017) compared with males. In contrast, males presented a 6% greater use of morphine (10% vs. 4% in females, *p* = 0.005) and a 13% of neuromodulators prescription (54% vs. 41%, *p* = 0.002).

In cases, both sexes doubled their MEDD (120–163 mg/day, *p* < 0.001) compared to controls. As seen in Figure 2, they also presented a 17–15% higher buprenorphine and 12–14% benzodiazepines prescription (females: OR 1.62 [1.02–2.57] and males: OR 2.19 [1.15–4.17]). In contrast, a 24% lower use of tramadol (13% vs. 37% in controls, *p* < 0.001) was shown in female cases and a 22% greater fentanyl use (40% vs. 18%, *p* = 0.002; OR 2.97 [1.52–5.81]) was observed in male cases compared to controls.

### 3.3. Safety Outcomes

Analgesic drug tolerability is shown in Table 5 and Figure 2.

Women presented a higher number of AEs in both groups, being the highest (median of 6 AEs/patients) in female cases. In fact, ADRs were significantly greater in female controls than men (18% vs. 12%, *p* = 0.035). On the contrary, although male cases presented the lowest number of AEs (3 (1–6) AEs/patient), they doubled the male controls’ ADRs (24% vs. 12%, *p* = 0.039; OR 2.40 [1.12–5.16]).

Among reported cases, females presented 23% more dizziness (43% vs. 20% in males, *p* = 0.038), 17% edema (17% vs. 0%, *p* = 0.014), and 25% nervousness (52% vs. 27%, *p* = 0.026) compared to males. They suffered 17% more sleep disturbance compared to controls (52% vs. 35% in female controls, *p* = 0.012; OR 2.00 [1.18–3.42]). Meanwhile, in male reported cases, 16% higher vomiting (20% vs. 4%, *p* = 0.005; OR 5.58 [1.83–17.05]) and 18% itching (33% vs. 15%, *p* = 0.022; OR 2.77 [1.19–6.46]) rates were observed compared to controls. On the other hand, in the control group, females suffered 5% more vomiting (9% vs. 4% in males, *p* = 0.036), 17% weight change (36% vs. 19% males, *p* < 0.001), 14% dry skin (38% vs. 24% males, *p* < 0.001), 12% depression (35% vs. 23% males, *p* = 0.002), and 12% loss of appetite (28% vs. 16% males, *p* = 0.001) rates relative to males. Only sexual dysfunction was higher in males (25%) in comparison with females (17%, *p* < 0.001).

## 4. Discussion

Younger age, work disability, opioid doses higher than 120 mg/day of morphine equivalent daily dose and benzodiazepine use were found significantly higher in cases compared to controls (similarly for both sexes). Nevertheless, sex-differences in cases were found related to prior SUD, opioid prescription and tolerability. Thus, the use and dose of opioids should be carefully monitored in patients with these underlying factors.

Our data have shown known socio-economic risk factors along with co-medication of high doses of opioids and benzodiazepines [23,24]. However, sex-based differences were observed due to prior SUD [25] and fentanyl prescription [26]. Women have been described to report greater receipt of prescriptions for anxiolytics, sedatives or hypnotics, what could contribute to an OUD [27]. Other clinical evidence suggest that men are more sensitive than women to the abuse-related effects of mu-opioid agonists, as fentanyl, although preclinical studies differ from this evidence [28]. These findings support sex-based tailoring of treatment, but any tailoring should also consider person-level differences [29].

Greater and different pain treatment intensity in females might be a consequence of the normalization of pain symptom between women and family doctors, which may also lead to delays in pain diagnosis or referrals to a PU [30]. In this way, these potential diagnosis and therapeutics delays should be deeply analyzed in terms of biopsychosocial mechanisms, adjusting for confounding by gender, as they may underlie these sex-differences, and considerations for future research should be discussed [31,32]. Besides, among the psychosocial risks that can worsen the state of health of women, we find a significant higher dedication to household tasks. The physical discomfort caused by the overload of domestic work, as well as the physical and mental stress resulting from the double working day as employees and as caregivers for the whole family, calls for further studies to identify appropriate intervention and prevention strategies [33].

The worse analgesic tolerability in females -related to gastrointestinal and nervous systems- and 10% more emergency department visits, falls in line with previously published scientific literature [34,35,36,37]. However, in spite of male cases having the lowest number of AEs, they arose the highest ADRs notification. These sex-differences are not fully elucidated [38]. Possible multifaceted factors seem to be associated. These include neuroanatomical, hormonal, neuroimmunological, but also psychological plus other social and cultural factors which need to be deeper analyzed (along with a gender perspective). In this way, studies should no longer consider men and women as a homogeneous group, given that subjective painkillers’ tolerability substantially differs between sexes [17,39].

The exploratory nature of the study has permitted us to establish differences between women and men in some intersectional factors such as age. However, it did not allow us to collect essential information that would enable us to link, for example, whether this fact was caused by a delayed diagnosis in women [40,41]. For this reason, it is essential to consider potential gender stereotypes threats that could affect our varied experiences and overall health [42].

There are some limitations in this study that need to be acknowledge. First, the sample size was limited by a “convenience sample” due to the low incidence of OUD. Secondly, although the control group came from the same setting, it was composed by subjects from different studies. Moreover, most patients were under other non-opioid centrally acting drugs related to their diverse comorbidities, which might have independently contributed to the observed side-effects. This could introduce a bias mediated by several other variables, such as socio-demographics, that could be more relevant than pain status [43,44]. What’s more, the higher prevalence of buprenorphine among cases could be part of the beginning of medication assisted therapy, prior to the derivation to the PU for the opioid tapering procedure. The data collection of some variables such as prior SUD could have been limited by the poor documentation from healthcare professionals. Nevertheless, this information was gathered through medical diagnoses, narratives, and Addictive Behavior Unit visits. All in all, this study helps to create more information about the needs of these patients to design more equal prevention programs.

## 5. Conclusions

In light of the above information, sex-differences in pain management and OUD risk have been observed. A deeper analysis of sex-gender interactions may be needed to understand disparities in potential diagnosis delays, analgesic prescription, safety pattern and healthcare resources use. Hence, further research is needed to refine these results and explore potential gender disparities in order to optimize individual pain and OUD management.

## Figures and Tables

**Table 1:** Socio-demographic analysis by sex.

	Women		Men		Women	Men	Controls	Cases
	**Controls** **442 (66%)**	**Cases** 88 (64%)	**Controls** 227 (34%)	**Cases** 49 (36%)	♀^CONTROLS^ vs. ♀^CASES^ *	♂^CONTROLS^ vs. ♂^CASES^ *	♀^CONTROLS^ vs. ♂^CONTROLS^	♀^CASES^ vs. ♂^CASES^ *
Age (years old) (med (IQR))	66 (56–75) *	53 (45–65)	60 (49–73)	53 (45–61)	<0.001 **0.074**	<0.001 0.042	0.001 0.018	0.636 0.087
Employment status (%)								
Active	19	6	13	4	0.022 0.136	0.191 0.127	0.273 0.067	1.000 0.050
Retired	56	22	53	28	<0.001 **0.277**	0.032 0.199	0.550 0.039	0.584 0.079
Work disability	9	41	22 *	64	<0.001 **0.229**	0.009 **0.242**	0.003 0.168	0.062 **0.221**
Unemployed	9	4	12	0	0.271 0.075	0.070 0.164	0.555 0.036	0.537 0.120
Homemaker	7 *	27 *	0	4	<0.001 **0.264**	0.212 0.168	0.004 0.148	0.014 **0.291**
Income (%)								
Less than €500	31	60	6	40	0.106 0.177	0.128 **0.411**	0.071 **0.285**	0.617 0.175
Between €500–1000	62	27	63	40	0.055 **0.336**	0.611 0.194	1.000 0	0.613 0.127
More than €1000	7	13	31	20	0.596 0.107	1.000 0.107	0.079 **0.322**	1.000 0.081

* The values for the *p*-value are shown first and then for the effect size. ^1^ In grey higher value for differences between controls vs. cases in women/men (*p*-value < 0.05). ^2^ * *p*-value < 0.05 for differences between women vs. men in controls/cases (higher value in bold). ^3^ Effect size: Eta-squared (η^2^ = 0.01 indicates a small effect; η^2^ = 0.06 indicates a medium effect; η^2^ = 0.14 indicates a large effect), Cohen’s D (small: 0.2, intermediate: 0.5, and large effect: 0.8) and Cramer’s V (small < 0.2, 0.2 < intermediate < 0.6, and large effect > 0.6). ^4^ From medium effect marked in bold.

**Table 2:** Clinical analysis by sex.

	Women		Men		Women	Men	Controls	Cases
	**Controls** **442 (66%)**	**Cases** 88 (64%)	**Controls** 227 (34%)	**Cases** 49 (36%)	♀^CONTROLS^ vs. ♀^CASES^ *	♂^CONTROLS^ vs. ♂^CASES^ *	♀^CONTROLS^ vs. ♂^CONTROLS^	♀^CASES^ vs. ♂^CASES^ *
Clinical Outcomes (mean (SD))								
Pain intensity (VAS, 0–100 mm)	60 (28)	60 (28)	57 (28)	59 (26)	0.999 0	0.909 0	0.319 0.002	0.753 0.001
Pain relief (VAS, 0–100 mm)	35 (29)	38 (30)	33 (29)	34 (29)	0.566 0.001	0.854 0	0.277 0.002	0.480 0.004
EQ (VAS, 0–100 mm)	44 (23)	42 (24)	45 (23)	50 (24)	0.364 0.002	0.302 0.004	0.654 0	0.116 0.021
Substance Use Disorder (SUD, %)								
Previous SUD	11	19	16	21	0.029 0.101	0.399 0.051	0.063 0.075	1.000 0.015
Tobacco	10	17	15	19	0.061 0.086	0.515 0.040	0.058 0.074	0.818 0.019
Alcohol	0.2	2	1	0	0.072 0.102	1.000 0.040	0.267 0.046	0.538 0.091
Illicit substances	0.5	0	0	2	1.000 0.028	0.175 0.131	0.551 0.039	0.356 0.116

* The values for the *p*-value are shown first and then for the effect size. ^1^ EQ: EuroQol scale (0–100 mm); VAS: Visual Analogue Scale (0–100 mm). ^2^ In grey higher value for differences between controls vs. cases in women/men (*p*-value < 0.05). ^3^ * *p*-value < 0.05 for differences between women vs. men in controls/cases (higher value in bold). ^4^ Effect size: Eta-squared (η^2^ = 0.01 indicates a small effect; η^2^ = 0.06 indicates a medium effect; η^2^ = 0.14 indicates a large effect), Cohen’s D (small: 0.2, intermediate: 0.5, and large effect: 0.8) and Cramer’s V (small < 0.2, 0.2 < intermediate < 0.6, and large effect > 0.6). ^5^ From medium effect marked in bold.

**Table 3:** Analgesic analysis by sex.

	Women		Men		Women	Men	Controls	Cases
	**Controls** **442 (66%)**	**Cases** 88 (64%)	**Controls** 227 (34%)	**Cases** 49 (36%)	♀^CONTROLS^ vs. ♀^CASES^ *	♂^CONTROLS^ vs. ♂^CASES^ *	♀^CONTROLS^ vs. ♂^CONTROLS^	♀^CASES^ vs. ♂^CASES^ *
Simple analgesics	45 *	37	37	40	0.157 0.065	0.741 0.026	0.039 0.081	0.712 0.036
Tramadol	37 *	13	25	25	<0.001 0.194	1.000 0	0.001 0.128	0.093 0.157
MEDD (mg/day) (med (IQR))	60 (40–120)	120 (60–200)	60 (40–116)	163 (80–250)	<0.001 0.049	<0.001 **0.078**	0.853 0	0.313 0.008
Fentanyl	19	28	18	40	0.083 0.077	0.002 0.198	0.755 0.014	0.179 0.123
Oxycodone	35	36	42	29	0.903 0.006	0.106 0.102	0.064 0.073	0.568 0.066
Tapentadol	35	30	29	17	0.390 0.039	0.106 0.106	0.140 0.058	0.102 0.146
Buprenorphine	3	20	4	19	<0.001 **0.266**	0.001 **0.227**	0.497 0.027	1.000 0.009
Morphine	4	8	10 *	6	0.160 0.069	0.587 0.046	0.005 0.112	1.000 0.033
Hydromorphone	1	1	0.4	0	1.000 0.006	1.000 0.028	0.433 0.043	1.000 0.064
Immediate release opioids	18	15	19	17	0.642 0.025	0.840 0.018	0.805 0.018	0.671 0.027

* The values for the *p*-value are shown first and then for the effect size. ^1^ MEDD: morphine equivalent daily dose. ^2^ In grey higher value for differences between controls vs. cases in women/men (*p*-value < 0.05). ^3^ * *p*-value < 0.05 for differences between women vs. men in controls/cases (higher value in bold). ^4^ Effect size: Eta-squared (η^2^ = 0.01 indicates a small effect; η^2^ = 0.06 indicates a medium effect; η^2^ = 0.14 indicates a large effect), Cohen’s D (small: 0.2, intermediate: 0.5, and large effect: 0.8) and Cramer’s V (small < 0.2, 0.2 < intermediate < 0.6, and large effect > 0.6). ^5^ From medium effect marked in bold.

**Table 4:** Pharmacological and health use analysis by sex.

	Women		Men		Women	Men	Controls	Cases
	**Controls** **442 (66%)**	**Cases** 88 (64%)	**Controls** 227 (34%)	**Cases** 49 (36%)	♀^CONTROLS^ vs. ♀^CASES^ *	♂^CONTROLS^ vs. ♂^CASES^ *	♀^CONTROLS^ vs. ♂^CONTROLS^	♀^CASES^ vs. ♂^CASES^ *
NSAIDs	16	14	16	15	0.632 0.026	1.000 0.015	1.000 0	1.000 0.015
Neuromodulators	41	48	54 *	60	0.235 0.055	0.521 0.044	0.002 0.122	0.277 0.108
Antidepressants	42 *	51	31	36	0.158 0.062	0.499 0.040	0.006 0.107	0.146 0.134
Benzodiazepines	41 *	53	27	45	0.044 0.089	0.022 0.146	<0.001 0.137	0.469 0.078
Health Resources Use data (%)								
Emergency department visits	32 *	22	22	28	1.000 0	0.217 0.105	0.017 0.106	0.768 0.069
Hospitalisation	5	3	5	5	1.000 0.034	1.000 0	1.000 0.010	1.000 0.056
Medication changes	31	32	29	35	0.848 0.009	0.606 0.043	0.677 0.022	1.000 0.027

* The values for the *p*-value are shown first and then for the effect size. ^1^ NSAIDs: non-steroidal anti-inflammatory drugs. ^2^ In grey higher value for differences between controls vs. cases in women/men (*p*-value < 0.05). ^3^ * *p*-value < 0.05 for differences between women vs. men in controls/cases (higher value in bold). ^4^ Effect size: Eta-squared (η^2^ = 0.01 indicates a small effect; η^2^ = 0.06 indicates a medium effect; η^2^ = 0.14 indicates a large effect), Cohen’s D (small: 0.2, intermediate: 0.5, and large effect: 0.8) and Cramer’s V (small < 0.2, 0.2 < intermediate < 0.6, and large effect > 0.6). ^5.^ From medium effect marked in bold.

**Table 5:** Safety variables description by sex.

	Women		Men		Women	Men	Controls	Cases
	**Controls** 413 (66%)	**Cases** 63 (68%)	**Controls** 210 (34%)	**Cases** 30 (32%)	♀^CONTROLS^ vs. ♀^CASES^ *	♂^CONTROLS^ vs. ♂^CASES^ *	♀^CONTROLS^ vs. ♂^CONTROLS^	♀^CASES^ vs. ♂^CASES^ *
Adverse Drug Reactions	18 *	15	12	24	0.541 0.035	0.039 0.138	0.035 0.083	0.173 0.121
Adverse Events (med (IQR))	5 (2–8) *	6 (3–8) *	4 (2–6)	3 (1–6)	0.335 0.002	0.547 0.001	0.003 0.014	0.042 0.044
Sleepiness	38	41	38	23	0.580 0.026	0.156 0.099	1.000 0	0.109 0.175
Dizziness	33	43 *	26	20	0.155 0.069	0.653 0.044	0.066 0.076	0.038 **0.223**
Nausea	23	29	17	27	0.346 0.042	0.212 0.081	0.097 0.071	1.000 0.021
Vomiting	9 *	10	4	20	1.000 0.005	0.005 **0.215**	0.036 0.088	0.192 0.146
Constipation	51	40	48	37	0.104 0.079	0.328 0.076	0.447 0.031	0.823 0.029
Itching	21	25	15	33	0.417 0.036	0.022 0.157	0.105 0.069	0.464 0.083
Sexual dysfunction	8	5	25 *	13	0.603 0.039	0.247 0.089	<0.001 **0.236**	0.207 0.152
Loss of libido	20	21	27	33	0.865 0.007	0.510 0.051	0.087 0.087	0.206 0.138
Weight change	36 *	40	19	20	0.575 0.027	0.806 0.013	<0.001 0.178	0.065 0.195
Headache	32	33	26	27	0.885 0.010	1.000 0	0.141 0.060	0.634 0.067
Skin redness	17	6	16	13	0.035 0.110	1.000 0.023	0.704 0.021	0.267 0.116
Dry skin	38 *	35	24	20	0.677 0.023	0.818 0.030	<0.001 0.145	0.157 0.152
Dry mouth	61	59	54	37	0.783 0.015	0.117 0.114	0.103 0.067	0.075 **0.206**
Edema	13	17 *	11	0	0.432 0.041	0.089 0.123	0.444 0.034	0.014 **0.253**
Depression	35 *	40	23	23	0.482 0.033	1.000 0	0.002 0.124	0.162 0.161
Sleep disturbance	35	52	28	47	0.012 0.119	0.053 0.138	0.058 0.079	0.661 0.054
Nervousness	42	52 *	35	27	0.135 0.070	0.415 0.060	0.101 0.067	0.026 **0.242**
Loss of appetite	28 *	33	16	20	0.455 0.039	0.603 0.034	0.001 0.132	0.227 0.137

* The values for the *p*-value are shown first and then for the effect size. ^1^ In grey higher value for differences between controls vs. cases in women/men (*p*-value < 0.05). ^2^ * *p*-value < 0.05 for differences between women vs. men in controls/cases (higher value in bold). ^3^ Effect size: Eta-squared (η^2^ = 0.01 indicates a small effect; η^2^ = 0.06 indicates a medium effect; η^2^ = 0.14 indicates a large effect), Cohen’s D (small: 0.2, intermediate: 0.5, and large effect: 0.8) and Cramer’s V (small < 0.2, 0.2 < intermediate < 0.6, and large effect > 0.6). ^4^ From medium effect marked in bold.

## Data Availability

The data presented in this study are available on request from the corresponding author. The data are not publicly available due to confidentiality concerns of the data.
